# Vicarious Menstruation from the Gums

**Published:** 1900-03

**Authors:** W. George Beers


ARTICLE IX.

### VICARIOUS MENSTRUATION FROM THE GUMS.

W. GEORGE BEERS.

Since last January 1, 1900, I have had a very complicated case of contraction of the superior and inferior teeth to contend with, and during the frequent visits of the patient, aged seventeen, I had opportunity for observing one of the most interesting cases of vicarious menstruation which the limitations of dental practice has ever brought to me. The day after I had taken the impression for models, the patient came by appointment, and I noticed such an effusion of blood, about the gingival margins especially, and the gums generally, that at first I suspected that the blood in the mouth came from the lungs. But upon careful observation there was no mistake about the matter, and the surprise was the greater because the gums were healthy and the teeth free from caries or calculus. A week afterward I inserted the apparatus for expanding the superior arch. It was worn with comfort until the periodical return of the menses, when the margins of the gums—which the plate did not touch—were inflamed, as if by the rough inner edges of a badly-fitting vulcanite plate, and the bleeding reappeared. Upon examination of the apparatus there was no exciting mechanical cause to produce such a result, and I was at a loss for an explanation until I found that the blood was non-coagulable, and that the same symptoms of hysteria were present which I had observed the previous month. I then made it my duty to extend my inquiries, and learned that the condition was regularly present each month, associated with severe migraine, and that the bleeding began and ceased coincidently with the recurrence and cessation of menstruation. As I write, the ninth observation has been made.—*Dominion Dental Journal.*

